# Many-Body Study of Iron(III)-Bound Human Serum Transferrin

**DOI:** 10.1021/acs.jpclett.2c00680

**Published:** 2022-05-12

**Authors:** Hovan Lee, Cedric Weber, Edward B. Linscott

**Affiliations:** † Department of Physics, Faculty of Natural & Mathematical Sciences, King’s College London, London WC2R2LS, U.K.; ‡ Theory and Simulation of Materials (THEOS), École Polytechnique Fédérale de Lausanne, 1015 Lausanne, Switzerland

## Abstract

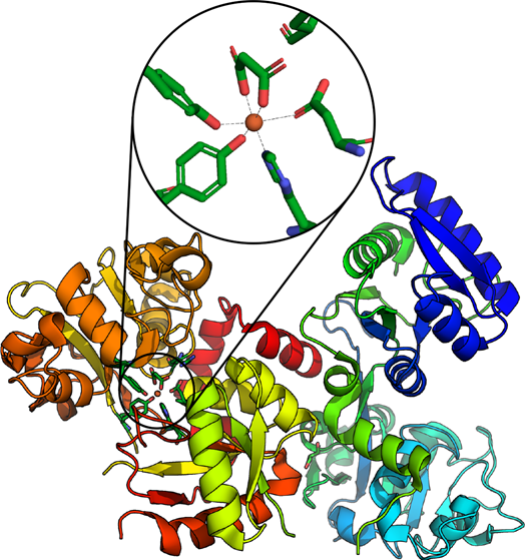

We present the very first density functional theory and dynamical mean field theory calculations of iron-bound human serum transferrin. Peaks in the optical conductivity at 250, 300, and 450 nm were observed, in line with experimental measurements. Spin multiplet analysis suggests that the ground state is a mixed state with high entropy, indicating the importance of strong electronic correlation in this system’s chemistry.

Metabolism and regulation of iron play a fundamental role in the homeostasis of the vast majority of living organisms. Vertebrates in particular are reliant on iron, primarily due to the need to synthesize hemoglobin and myoglobin for oxygen transport in blood and oxygen storage in muscle cells, respectively. Furthermore, an imbalance of iron leads to an extensive number of health problems such as anemia (from iron deficiency) and arrhythmia (from iron overload) among others.^1−6^

Facilitating iron regulation in vertebrates are the transferrins, a group of metal binding glycoproteins that mediate the transport of iron through blood plasma. These glycoproteins are formed from single polypeptide chains with molecular weights of ∼80 kDa, each containing two metal binding sites.

Crystallographic studies of human serum transferrin (hTF) revealed folded lobes at both the carboxyl and amino termini of the polypeptide chain (termed the C-lobe and N-lobe, respectively). These lobes contain identical metal binding sites, insofar as the coordination complex amino acids are involved. Each site consists of two tyrosine residues, one histidine residue, and one aspartic acid residue, with an additional synergistic bidentate anion (such as a carbonate or a malonate ion) to complete the octahedral metal complex, as shown in the abstract graphic.

Over the past several decades, a substantial effort has been made to investigate the metal binding mechanism of hTF. Recent endeavors in the X-ray diffraction of crystallized hTF include structures bound with Ti(IV) (both lobes),^7^ Yb(III) (C-lobe),^8^ and Cr(III) (C-lobe).^9^ These works have elucidated the conditions and ligand bond distances needed to accommodate the binding of various ions.

The conformational changes in hTF have also been studied computationally, using classical molecular dynamics. The latest endeavors include an investigation of the effects of various synergistic and nonsynergistic anions on the stability of the binding configuration^10^ and an analysis of the effect of pH-induced changes on the conformation and its link to the binding/release mechanism of hTF.^11^

However, classical molecular dynamics do not explicitly model the electrons, as is done in more accurate computational approaches such as Kohn–Sham density functional theory (DFT). DFT has been used to calculate the energies of binding of hTF with various ions. Sanna et al. investigated the different sites responsible for binding VO^2+^,^12^ while Justino et al. focused on the binding of VO^2+^ in the N-lobe.^13^ Sakajiri et al. simulated the binding energies of several different metal ions,^14^ and Reilley et al. examined the uptake and release of a variety of different metal ions.^15^

To calculate the properties of a molecule in DFT, the material is portrayed as an auxiliary system of non-interacting particles. Here the electrons have no explicit influence on one another and instead interact at the mean field level, whereby each electron experiences a local potential that approximates the many-body electron−electron Coulombic repulsion. This level of theory is sufficient for predicting the attributes of a large number of proteins. This is not the case, however, for systems in which localized many-body effects are important (such as those containing transition metals).^16−21^ Electrons in these systems are so close to one another that their interactions become too substantial to be treated through the approximation of exchange and correlation in DFT.

One approach for addressing this issue is to use hybrid functionals. These functionals have an exchange term that is a linear combination of semilocal DFT exchange and Hartree−Fock exchange. The mixing of the two can be carried out such that the problem of self-interaction is minimized. However, hybrid functionals are computationally expensive and scale poorly for large systems such as ∼80 kDa proteins. Moreover, hybrid functionals do not include any electronic correlation beyond that contained in the base DFT functional.

In this work, we apply dynamical mean field theory (DMFT)^22−26^ to study the binding site of hTF. DMFT is a Green’s function approach that explicitly calculates the many-body properties of interacting electrons. This approach has found previous success in strongly correlated many-body problems such as explaining the insulating M_1_ phase of vanadium dioxide,^27^ modeling the photodissociation of carboxymyoglobin,^28^ and correctly predicting the binding energies of myoglobin^29^ and hemoglobin^30^ through explicit inclusion of Hund’s coupling.

DFT+DMFT calculations were performed on three different cluster models of the hTF binding site: one structure with a synergistic malonate anion and two structures with carbonate anions (in this work termed structures CARB A and CARB B).

First, let us examine the spectroscopic properties of hTF as predicted by the DFT+DMFT calculations. The DFT+DMFT local density of states (LDOS) of the malonate structure is shown in Figure 1a. The density of states is decomposed into the contributions of the amino acid residues, the Fe ion, and the malonate ion. Several Fe-localized features can be seen below the Fermi level, which is consistent with our expectations. The electrons that are most susceptible to excitations are associated with those of the Fe 3*d* orbital, while those of the other atoms (H, C, N, and O) are more tightly bound and are expressed as wide bands below −1 eV. Above the Fermi level, the lowest unoccupied molecular orbital (LUMO) starting at ∼1 eV is delocalized throughout the binding site.

**Figure 1:**
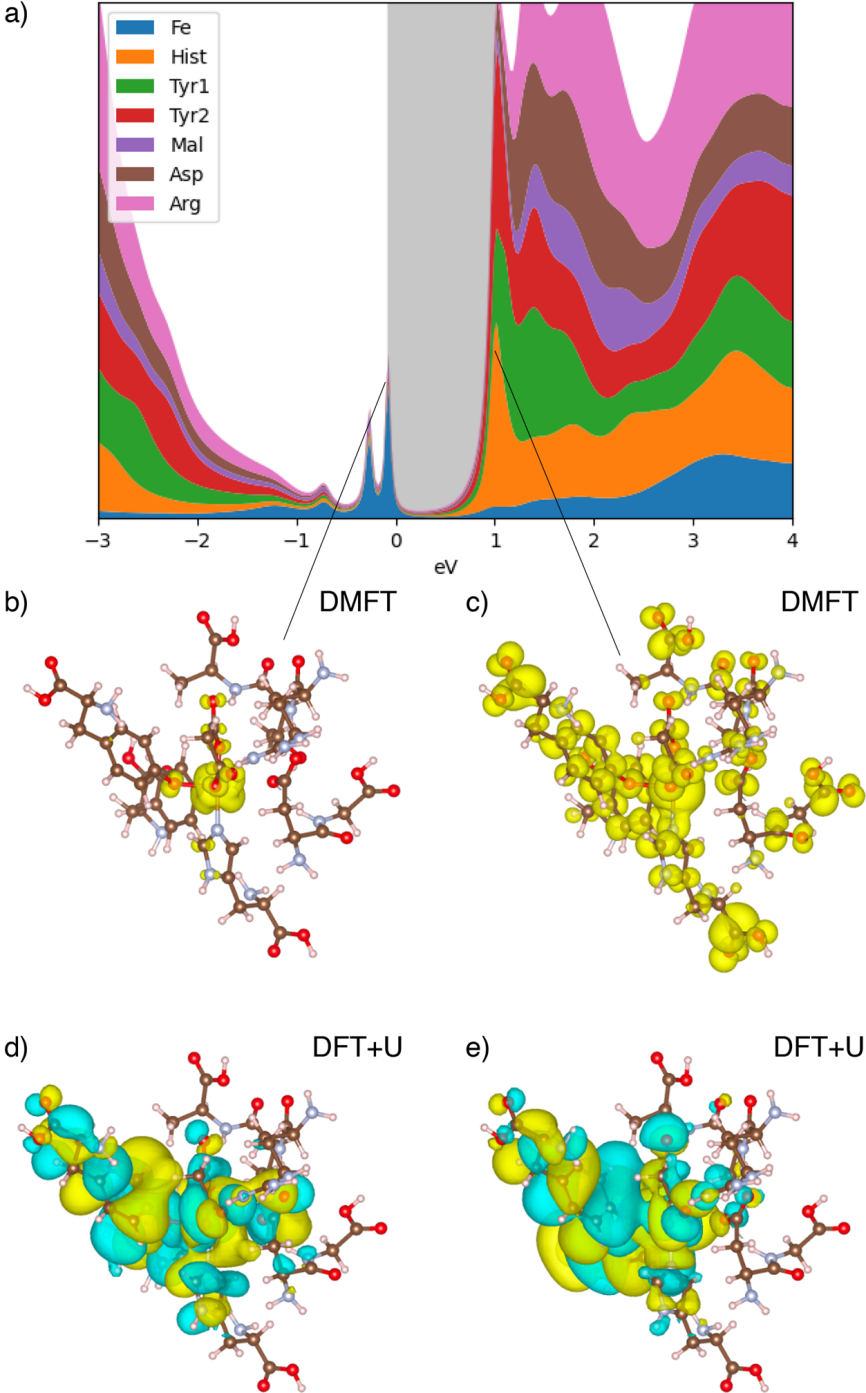
(a) Density of states (DOS) of the malonate structure, ranging from −3 to 4 eV, resolved spatially into the hydrogenic orbitals of the amino acid residues, the Fe ion, and the malonate ion. We observe delocalized, high-amplitude features in the DOS ranging from −3 to −1.5 eV. Between −1 eV and the Fermi level, we observe Fe-localized peaks in the LDOS. The lowest unoccupied molecular orbital (LUMO) and other delocalized higher-energy features are seen from ∼1 eV onward. The real-space resolved DFT+DMFT density of the highest occupied molecular orbital (HOMO) and the LUMO are illustrated in panels b and c, respectively. The DFT+*U* spin = 5ℏ/2 HOMO and LUMO Kohn−Sham orbitals are depicted in panels d and e, respectively, where yellow (blue) areas indicate positive (negative) parts of the wave function.

The DFT+DMFT electronic densities of the highest occupied molecular orbital (HOMO) and the LUMO are shown in panels b and c, respectively, of Figure 1. For comparison, we also show the analogous Kohn−Sham wave functions for a DFT+*U* calculation with broken-spin symmetry and a total spin of 5ℏ/2. (The energy of this state is lower than those of the ℏ/2 and 3ℏ/2 alternatives.) The DFT+DMFT HOMO is localized around the Fe ion, and the LUMO is delocalized throughout the binding site. This picture is qualitatively different from what DFT+*U* predicts, where the HOMO and LUMO are both delocalized throughout the binding site. Clearly, strong local electronic interactions, as included in DMFT but not in DFT+*U*, drive the localization of the HOMO.

Next, we present the optical conductivity spectra of all three hTF structures. These were obtained by applying Kubo−Greenwood relations to the DFT+DMFT density of states. The isotropic optical conductivity σ(ω), defined as the average of the diagonal elements of the frequency-dependent optical conductivity matrix, is depicted in Figure 2 for the malonate (red), carbonate A (green), and carbonate B (blue) structures. A separate isotropic optical conductivity calculation with DFT+*U* (dashed black) was also carried out for the malonate structure. All three structures concur with the experimentally observed ultraviolet−visible spectroscopy bands: sharp features at 254 and 298 nm and a wide band at 470 nm.^31^ With DFT+*U*, the feature at ∼300 nm is reduced, and the underlying broadband spectra vanish at ∼450 nm. The loss of these ultraviolet-visible features suggests an improper characterization of the correlated 3*d* orbital of the Fe ion.

**Figure 2:**
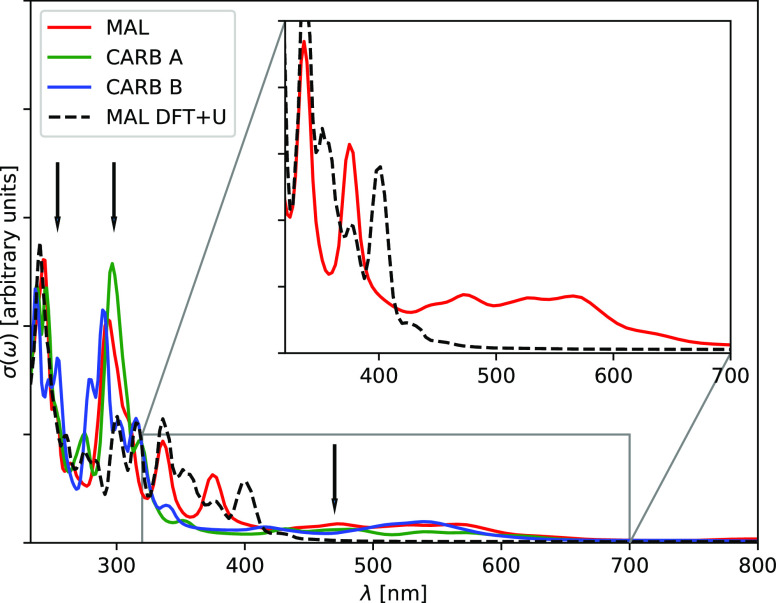
Isotropic optical conductivity, resolved in wavelength, depicted for the malonate (red), carbonate A (green), and carbonate B (blue) structures. We observe large amplitude features below 250 nm and at 300 nm, and the spectra extend up toward ∼700 nm. The isotropic optical conductivity for a DFT+*U* calculation of the malonate structure (dashed black) is also shown. Arrows depict experimentally observed ultraviolet−visible spectroscopic optical absorption bands at 254, 298, and 470 nm.^31^

To summarize, DFT+DMFT predicts spectroscopic properties of hTF that match very well with experiments. However, there is an underlying degree of freedom in the DMFT calculations that we have not yet discussed. This is the double-counting parameter.^32^

The DFT part of these DFT+DMFT calculations treats electronic correlations with delocalized exchange-correlation functionals. The chosen DMFT localized subspace (in this instance, the 3*d* orbital of iron) is then augmented with screened Coulomb interaction and Hund’s coupling explicitly. This calls for a double-counting term to correct the component of correlation already included within DFT.

The results of any DFT+DMFT calculation are sensitive to the value of this double-counting parameter. One can in principle determine this parameter from first principles, but we can also treat it as a free parameter that allows us to artificially control the charge of the iron ion. This allows us to explore the electronic state of the iron site in great detail.

It is important to note that this double-counting parameter is not wholly disconnected from physical processes. As we will see, the double-counting parameter affects the occupancy of the iron 3*d* orbital, similarly to what would happen when the iron−ligand bond distances change. In the physical system, the iron−ligand bond distances will vary (e.g., due to thermal fluctuations or variations in pH), and thus, the system will effectively explore the immediate neighborhood of “double-counting space”.

We investigate the effects of this double-counting parameter in Figure 3 and report the iron 3*d* orbital occupancy for all three structures in terms of hydrogenic electron counting: Fe(III) + Δ, where Fe(II) = Fe(III) + 1. For all three structures, the 3*d* orbital occupation increases with double counting *n*_d_, but the two parameters are not directly equivalent: a given double counting tends to result in a slightly larger 3*d* orbital occupancy. For example, an *n*_d_ of 3.0 results in approximately Fe(III) – 0.25, equating to ∼4.75 3*d* electrons. In our view, this is due to ligand charge donation: the reshaping of ligand electron wave functions upon binding, causing a higher value of electron density around the ion. Nevertheless, the general trend is clear: with a change in the double-counting parameter, the occupancy of the iron site—and, more generally, its electronic state—will change. We also note that the earlier DFT+DMFT results of Figures 1 and 2 used a double-counting parameter that yields the closest 3*d* occupation to the DFT+*U* converged value of approximately Fe(III) + 0.8. This does not mean that here we are discarding the benefits afforded to us by the use of DFT+DMFT. As a mean field theory, DFT+DMFT is not expected to induce large changes in local occupancies. Instead, the greatest advantages of DFT+DMFT are its ability to capture multideterminant solutions, low-energy excitations, and quasi-particle lifetimes, all of which are important for a proper description of optical spectra and magnetic states.

**Figure 3:**
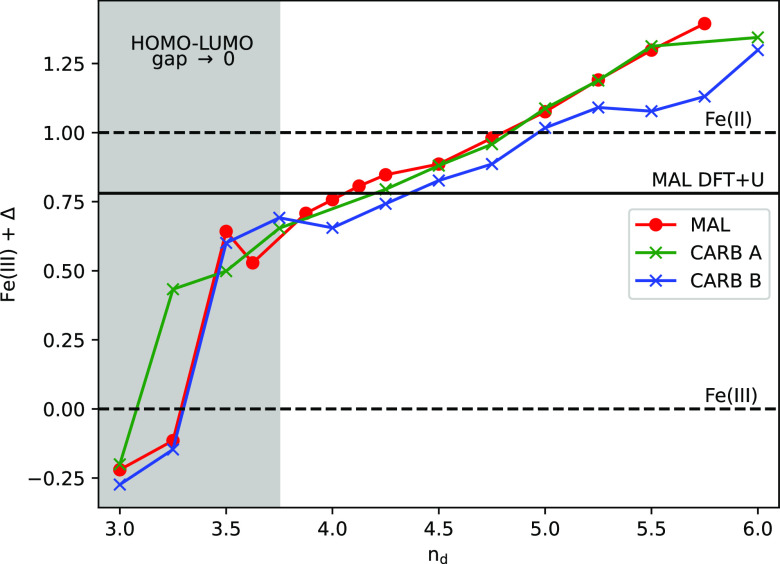
Electronic occupation of the iron 3*d* orbital, shown as Δ, the difference in occupation from Fe(III), for a range of double counting values as applied in the DMFT calculations. The reference Fe(III) and Fe(II) electronic occupations are shown as horizontal dashed lines. A solid horizontal line depicts the DFT+*U* 3*d* orbital filling calculated with a system spin of 5ℏ/2. The resultant range of occupation spans from approximately Fe(III) – 0.25 to approximately Fe(III) + 1.4 electrons. The gray region illustrates the DFT+DMFT converged solutions where the gap of the system tends toward zero.

Further exploiting the freedom afforded to us by the double-counting parameter, we explore its effects on the effective spin values of the system in Figure 4. The effective spin is implicitly defined via the equation , where ρ̂ is the projected 3*d* orbital electronic density matrix and *Ŝ* is the spin operator. In all three structures, we observe plateaus of decreasing *S*_eff_ as the 3*d* orbital occupancy increases.

**Figure 4:**
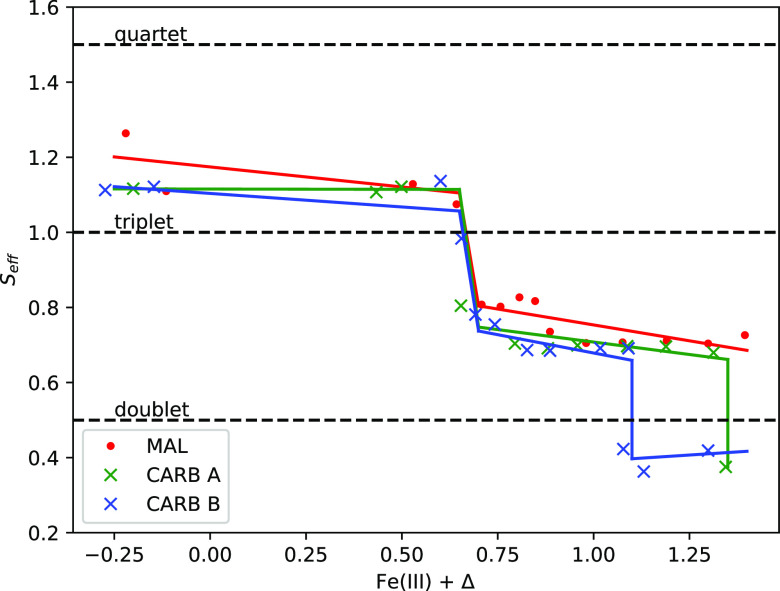
Effective spin value *S*_eff_, defined via Tr[] = ℏ^2^*S*_eff_(*S*_eff_ + 1), over the range of 3*d* orbital electronic occupation considered. Horizontal dashed lines are depicted as references to the doublet, triplet, and quartet spin states. For all three structures, we observe *S*_eff_ values of ∼1.1 from approximately Fe(III) – 0.25 to approximately Fe(III) + 0.65. In the case of the malonate structure (red dots), *S*_eff_ decreases to 0.8 at Fe(III) + 0.7 and continues in a declining plateau to a value of ∼0.7 up to Fe(III) + 1.4. The *S*_eff_ trend of the carbonate A structure (green crosses) is similar to that of the malonate structure, with an additional data point at approximately Fe(III) + 1.3 (*S*_eff_ ∼ 0.4), suggesting the onset of a further decrease in *S*_eff_. *S*_eff_ of the carbonate B structure (blue crosses) is similar to that of the other structures, with the addition of a second decrease and a plateau of *S*_eff_ at a value of ∼0.4 spanning from approximately Fe(III) + 1.1 to approximately Fe(III) + 1.3. Straight lines are a guide to emphasize the plateaued effective spin values.

We note in Figure 3 that the DFT+*U* calculation converged to a 3*d* occupancy of approximately Fe(III) + 0.8 and that the DFT+DMFT solutions of <Fe(III) + 0.7 converge to unphysical electronic structures with a negligible HOMO–LUMO gap. Therefore, we surmise that the *S*_eff_ ∼ 0.7 plateau corresponds to the physically meaningful subset of results.

This *S*_eff_ value of 0.7 and the *S*_eff_ value of other plateaus do not neatly align with the half-integer values of pure multiplet states. Therefore, as a final effort to further characterize the electronic state of the iron in hTF, we expanded the ground state of the malonate structure calculations in terms of their multiplet contributions (Figure 5). We observe that over the range of 3*d* orbital occupancy, the ground states encompass singlet, doublet, triplet, and quartet basis states, while other multiplet states have negligible contributions. The entropy associated with the multideterminant ground states is also depicted as black dots and a dashed black line. At approximately Fe(III) + 0.7, we see a sharp change in the multiplet contributions and a simultaneous peak in the entropy. This increased entropy indicates that an increased number of electronic configurations contribute to the electronic state of the 3*d* orbital near this transition.

**Figure 5:**
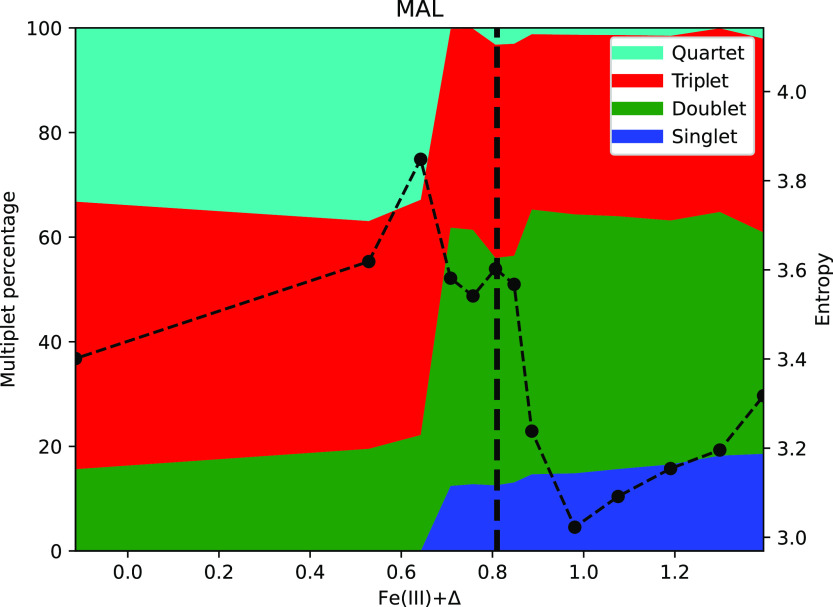
Multiplet analysis of the ground states of the malonate structure calculations, ranging from Fe(III) – 0.1 to Fe(III) + 1.4, resolved in the percentages of different contributing multiplet basis states. We remark that the ground states of the calculations from Fe(III) – 0.1 to Fe(III) + 0.65 consist of ∼20% doublet, ∼40% triplet, and ∼40% quartet states. A sharp spin switch occurs as the electronic occupation increases, and the ground states of the calculations from Fe(III) + 0.7 to Fe(III) + 1.4 consist of ∼20% singlet, ∼40% doublet, ∼40% triplet, and <5% quartet states. A dashed vertical line indicates the value of 3*d* occupancy used in Figures 1 and 2. Black dots with dashed line illustrate the entropy associated with the multideterminant state of the calculations.

There is one important caveat to keep in mind when interpreting these results. These values of entropy are obtained from a projected representation of the many-body electronic state onto the 3*d* orbital of the iron. Therefore, even if the full system existed in a pure state with zero entropy, the projection of this pure state could result in a projected density matrix with non-zero entropy. In other words, the reported entropy will include contributions from two sources: (a) entropy associated with the genuinely correlated nature of the 3*d* orbital and (b) entropy arising from the projection.

To disentangle these two potential sources of entropy, we calculated the von Neumann entropy of the single-determinant DFT+*U* 3*d* density matrix to be 1.4 (compare this to the DMFT entropies that range from 3.0 to 3.9 reported in Figure 5). Because the DFT+*U* solution is a single-Slater determinant, the entropy of the full solution is zero, and thus this entropy of 1.4 arises entirely from the entanglement between the projected subspace and the rest of the system. The DFT+*U* and DFT+DMFT entropies were calculated via two different approaches, so one should be cautious when comparing the two quantitatively; however, it appears that there are contributions to the DMFT entropy beyond that of hybridization with the rest of the system.

Crucially, if we focus on the physically meaningful value of the double counting [Fe(III) + 0.8, indicated with a vertical dashed line], we can conclude that the spin state comprises singlet, doublet, triplet, and quartet contributions and that the iron resides in a high-entropy region. This is a signature of strong electronic correlation.

Before closing, we should note that experimental measurements of iron-bound hTF, including Mössbauer^33,34^ and electron paramagnetic resonance (EPR) spectroscopy,^33,35,36^ produce results that indicate a high-spin Fe(III) sextet (*S*_eff_ = ^5^/_2_). From our perspective, the main contributing factor of the difference between our *S*_eff_ values and these experiments stems from the experimental requirement of low temperatures and high external magnetic fields; as an example, the EPR experiment^36^ was performed under external fields ranging from 7.5 to 12 T and at a temperature of 90 K. In comparison, our calculations were performed at 293 K in the absence of external magnetic fields, allowing us to identify the superposition of different multiplet states. This superposition of states would inevitably be disassembled as the temperature decreases and the external field strength increases (due to alignment of spins to the magnetic field), exciting the system out of its mixed state as it pertains to in vivo conditions, resulting in the experimentally observed *S*_eff_ = ^5^/_2_ state.

Indeed, an additional calculation of the Fe(III) + 0.8 system at a lower temperature (145 K) resulted in an electronic state comprised mostly of various sextet states (73%) and quintet states (27%), with an increased *S*_eff_ of 2.27ℏ compared to the 293 K *S*_eff_ value of ∼0.7ℏ. Crucially, this lower-temperature solution retains a large entropy: we observe only a 7% reduction of the entropy when going from 293 to 145 K. This suggests that the multitude of states that we observe is the result of not only temperature but also a multi-Slater-determinant ground state. Note that it is possible that our 293 K *S*_eff_ might also be affected by entanglement between the Fe ion and the rest of the system. As mentioned above, we have projected the density matrix, and much like for the entropy, this measure of the spin state *S*_eff_ can be affected by this projection.

In conclusion, we used DFT+DMFT to calculate the electronic properties of the transition metal binding site of human serum transferrin. We investigated three cluster models, and all three structures exhibit a tendency of increasing the electronic occupation of Fe(III) when it becomes bound to the binding site. The DFT+DMFT local density of states revealed a HOMO–LUMO gap of ∼1 eV, with the HOMO states localized at the Fe ion. The optical conductivities of the systems were calculated, with discernible features at 250, 300, and 450 nm, coinciding with the experimentally observed absorption peaks at 254, 298, and 470 nm. Further inspections of the multiplet contributions of the systems revealed that the ground states consist of singlet, double, triplet, and quartet states. Moreover, the system is in a high-entropy region. This indicates that strong electronic correlation plays an important role in the electronic state of iron in hTF.

## Supporting Information Available

Details of the DFT+*U* calculations, DFT+DMFT calculations, and associated quantum mechanical properties (PDF)
