# Supplementary material for: Many-Body Study of Iron(III)-Bound Human Serum Transferrin
Source: J Phys Chem Lett. 2022 May 12;13(20):4419–25. doi: 10.1021/acs.jpclett.2c00680 (PMC9150111; doi:10.1021/acs.jpclett.2c00680)
Supplement: Supplementary file 1 — jz2c00680_si_001.pdf [file jz2c00680_si_001.pdf]

Supporting Information: Many Body Study of Iron(III) Bound Human Serum Transferrin

Hovan Lee,*,† Cedric Weber,† and Edward B. Linscott*,‡

†*Department of Physics, Faculty of Natural & Mathematical Sciences, King's College London, London, WC2R2LS, UK*

‡*Theory and Simulation of Materials (THEOS), École Polytechnique Fédérale de Lausanne, 1015 Lausanne, Switzerland*

E-mail: hovan.lee@kcl.ac.uk; edward.linscott@epfl.ch

Crystallographic structures of carbonate (N-lobe) and malonate (C-lobe) Fe(III)-hTF were obtained from the Protein Data Bank archives[1] and[2] respectively. In the case of carbonate hTF, the crystallized orthorhombic ($P2_12_12_1$) structure contains alternative conformations of the synergistic carbonate anion, these structures are referred to in this work as structures A and B.

The binding sites of these structures were inspected, and cluster models were defined with a radius of 33Å, centered at the Fe ion. This resulted in a cluster of 149 atoms in the malonate structure, and 144 atoms in the carbonate structures. The clusters were then geometrically optimized, with the Fe ion, ligand donor atoms, and the amino acid backbone fixed.

To perform this geometry optimisation we used the linear-scaling DFT software ONETEP.[3,4] In ONETEP, DFT calculations are based on minimizing the system energy with respect to the single particle density matrix:

$$\rho(\mathbf{r},\mathbf{r'}) = \sum_{\alpha,\beta} \phi_\alpha(\mathbf{r}) K^{\alpha,\beta} \phi_\beta(\mathbf{r'}) \tag{1}$$

where $\{\phi_\alpha\}$ refers to a set of non-orthogonal generalized Wannier functions (NGWFs), and $K^{\alpha\beta}$ is the density kernel.

All DFT calculations were performed using the PBE XC functional,[5] with an energy cutoff at 900eV. There were thirteen NGWFs on the iron atom, four on each carbon, nitrogen, and oxygen, and one on each hydrogen. All NGWFs had a 6.5Å cutoff radius. A padded cell[6] of 34Å was applied to produce open boundary conditions. The systems were embedded in an implicit solvent with relative bulk permittivity of 80.[7] This approach avoids the spurious closing of the HOMO-LUMO gap that can occur in cluster models of proteins.[8]

Paramagnetic DFT+DMFT calculations were then performed on the optimized structure. This involves self-consistently calculating the Green's function as follows.

To start, a converged DFT calculation yields the NGWF basis Kohn-Sham Hamiltonian

$H^{\alpha\beta}$, from which the NGWF resolved Green's function is obtained:

$$G^{\alpha\beta}(\omega) = [(\omega + \mu)S - H - \Sigma(\omega)]^{-1}_{\alpha\beta} \tag{2}$$

where ω can be cast into $\omega + i\eta$ or $i\omega_n$ for broadening or finite-temperature Matsubara calculations, μ is the chemical potential, $S_{\alpha\beta}$ is the NGWF overlap matrix, and $\Sigma_{\alpha\beta}$ is the self energy.

In order to reintroduce the physics of strong correlation that is absent in DFT, the system is then downfolded onto an Anderson impurity model (AIM):

$$\hat{H} = \underbrace{\sum_{ij\sigma}(\varepsilon_{ij} - \mu)\hat{c}^{\dagger}_{i\sigma}\hat{c}_{j\sigma}}_{\hat{H}_{\text{bath}}} + \underbrace{\sum_{im\sigma}\left(V_{mi}\hat{f}^{\dagger}_{m\sigma}\hat{c}_{i\sigma} + h.c.\right)}_{\hat{H}_{\text{mix}}} + \underbrace{\sum_{mm'\sigma}(t_{mm'} - \mu)\hat{f}^{\dagger}_{m\sigma}\hat{f}_{m'\sigma} + \hat{H}_U}_{\hat{H}_{\text{loc}}} \tag{3}$$

where $\hat{H}_{bath}$ describes the non-correlated parts of the system (with hopping parameter ϵ_{ij}), $\hat{H}_{loc}$ characterizes the correlated orbitals by introducing the physics of strong correlation to the iron $3d$ subspace (with hopping parameter $t_{mm'}$ and Slater-Kanamori Hamiltonian $\hat{H}_U$) and $\hat{H}_{mix}$ represents the coupling in-between. From this, the non-interacting impurity Green's function is defined as:

$$G^0_{imp_{mm'}}(\omega)^{-1} = \omega + \mu - t - V_{mi}\left(\frac{1}{\omega + \mu - \varepsilon}\right)_{ij} V^{\dagger}_{jm'} \tag{4}$$

and the Green's function of the AIM

$$G_{imp_{mm'}}(\omega) = \left\langle \hat{f}_m \frac{1}{\omega - (\hat{H} - E_0)} \hat{f}^{\dagger}_{m'} \right\rangle + \left\langle \hat{f}^{\dagger}_{m'} \frac{1}{\omega + (\hat{H} - E_0)} \hat{f}_m \right\rangle \tag{5}$$

was calculated through the use of an exact diagonalization Lanczos solver.[9]

The impurity self-energy is then obtained via Dyson's equation:

$$\Sigma_{\text{imp}}(\omega) = [G^0_{\text{imp}}(\omega)]^{-1} - G^{-1}_{\text{imp}}(\omega) \tag{6}$$

This self-energy is then upfolded back into the NGWF basis, whereby properties such as the local density of states and the optical conductivity can be extracted.

The original DFT calculation already treats Coulomb interaction to some degree, we therefore need to apply corrections such that the Coulomb interactions are not double counted. n_d, as it is shown in Fig. 3 is accounted for as:

$$E_{DC} = \frac{U + 2(N-1)(U-2J)}{2N-1}(n_d - 0.5) + \frac{J}{2}(n_d - 1) \tag{7}$$

where N is the number of orbitals spanning the correlated subspace, U is the Hubbard U and J the Hund's exchange parameter. E_{DC} is the double counting correction to the impurity self-energy: $\Sigma_{imp,DC} = \Sigma_{imp} - E_{DC}$

The paramagnetic DFT+DMFT calculations presented in this work were performed using ONETEP and the DMFT package TOSCAM.[10] The calculations used a Slater-Kanamori Hubbard U and Hund's exchange J of 4.0eV and 0.8eV respectively, together with the inverse temperature value β of 39.6eV^{-1}. Seven bath sites, along with the CPT extension[11] were used in all DFT+DMFT calculations. The DFT+U results presented in Fig. 1 - 3 were calculated with a matching Hubbard parameter U_{eff} and exchange parameter J values of 4.0eV and 0.8eV respectively.[12–14] These calculations were spin-polarized with a total magnetization of $5\hbar/2$, corresponding to the lowest-energy spin configuration.

The iron $3d$ orbital occupation, as presented in Fig. 3 - 5 is obtained via projecting the DMFT converged ground state onto the Kohn-Sham solutions for a lone iron scalar relativistic pseudopotential generated with OPIUM.[15–22]

The effective spin (as presented in Fig. 4) is calculated via the reduced density matrix $\hat{\rho} = \sum_i e^{-\beta E_i} \text{Tr}_B[|i\rangle \langle i|]$, whereby a partial trace was taken over the bath degrees of freedom in the AIM. We can then define the effective spin of the iron atom, S_{eff}, via the expectation value of $\hat{S}^2 = \sum_{i,j} \hat{\mathbf{S}}_i \cdot \hat{\mathbf{S}}_j$ and the relation $\text{Tr}[\hat{S}^2 \hat{\rho}] = \hbar^2 S_{\text{eff}}(S_{\text{eff}} + 1)$.

The von Neumann entropy of the DFT+DMFT calculations (as presented in Fig. 5) is

given by:

$$S(\hat{\rho}) = -Tr[\hat{\rho}\ln(\hat{\rho})]. \tag{8}$$

In comparision, the DFT+U measure of von Neumann entropy[23] is obtained from the eigenvalues λ_k of the $3d$ orbital density matrix obtained from the Kohn-Sham solution:

$$\Lambda = -k_B \sum_k \lambda_k \ln(\lambda_k) \tag{9}$$

Multiplet contributions to the DMFT converged reduced density matrix (as presented in Fig. 5) are obtained from constructing the spin projector:

$$\hat{P}_S = \sum_{s \in S} |s\rangle \langle s| \tag{10}$$

where the eigenstates $|s\rangle$, when applied onto the spin operator $\hat{S}^2$, are associated with the eigenvalues $S(S+1)$. The fractions of the reduced density matrix, corresponding to different multiplet states, are obtained via $Tr[\hat{P}_S \hat{\rho} \hat{P}_S]$ for $S = 0, \frac{1}{2}, 1$ etc.

Local density of states (as presented in Fig. 1) is obtained from upfolding the converged DMFT impurity Hamiltonian into the NGWF basis. The NGWF basis resolved DFT+DMFT density matrix is then:

$$\rho^{\alpha\beta} = \frac{1}{2\pi i}(G^{\alpha\beta}(\omega) - G^{\alpha\beta\dagger}(\omega)) \tag{11}$$

and the trace of this density matrix can be taken to obtain the LDOS:

$$\rho_I(\omega) = \sum_{\alpha \in I} \sum_{\beta} \rho^{\alpha\beta}(\omega) S_{\alpha\beta} \tag{12}$$

where I denotes the subset of NGWFs that belong to each ion or amino acid residue.

The optical absorption spectrum of the system (as presented in Fig. 2) can be obtained

with the linear response Kubo formalism:

$$\sigma_{ij}(\omega) = \frac{2\pi}{\Omega} \int d\omega' \frac{f(\omega' - \omega) - f(\omega')}{\omega} \times \left(\rho^{\alpha\beta}(\omega' - \omega)\mathbf{v}^{i}_{\beta\gamma}\rho^{\gamma\delta}(\omega')\mathbf{v}^{j}_{\delta\alpha}\right) \tag{13}$$

where Ω is the simulation cell volume, $f(\omega)$ is the Fermi-Dirac distribution, ρ is the NGWF basis spectral density, i and j indices indicate Cartesian directions, the velocity operator $\mathbf{v}$ is defined as:

$$\mathbf{v}^{j}_{\alpha\beta} = -i \langle\alpha| \nabla_j |\beta\rangle + i \langle\alpha| \left[\hat{V}_{nl}, \mathbf{r}\right] |\beta\rangle \tag{14}$$

with non-local pseudopotentials $\hat{V}_{nl}$.

References

(1) Macgillivray, R.; Moore, S.; Chen, J.; Anderson, B.; Baker, H.; Luo, Y.; Bewley, M.; Smith, C.; Murphy, M.; Wang, Y. et al. Human Serum Transferrin, RECOMBINANT N-TERMINAL LOBE. 1998; `https://doi.org/10.2210/pdb1a8e/pdb`.

(2) Wang, M.; Zhang, H.; Sun, H. Human serum transferrin with ferric ion bound at the C-lobe only. 2015; `https://doi.org/10.2210/pdb4x1b/pdb`.

(3) Skylaris, C.-K.; Haynes, P. D.; Mostofi, A. A.; Payne, M. C. IntroducingONETEP: Linear-Scaling Density Functional Simulations on Parallel Computers. *J. Chem. Phys.* **2005**, *122*, 084119.

(4) Prentice, J. C. A.; Aarons, J.; Womack, J. C.; Allen, A. E. A.; Andrinopoulos, L.; Anton, L.; Bell, R. A.; Bhandari, A.; Bramley, G. A.; Charlton, R. J. et al. The ONETEP Linear-Scaling Density Functional Theory Program. *J. Chem. Phys.* **2020**, *152*, 174111.

(5) Perdew, J. P.; Burke, K.; Ernzerhof, M. Generalized Gradient Approximation Made Simple. *Phys. Rev. Lett.* **1996**, *77*, 3865–3868.

(6) Hine, N. D. M.; Dziedzic, J.; Haynes, P. D.; Skylaris, C.-K. Electrostatic interactions in finite systems treated with periodic boundary conditions: application to linear-scaling density functional theory. *J. Chem. Phys.* **2011**, *135*, 204103.

(7) Dziedzic, J.; Helal, H. H.; Skylaris, C.-K.; Mostofi, A. A.; Payne, M. C. Minimal parameter implicit solvent model for ab initio electronic-structure calculations. *EPL (Europhys. Lett.)* **2011**, *95*, 43001.

(8) Lever, G.; Cole, D. J.; Hine, N. D. M.; Haynes, P. D.; Payne, M. C. Electrostatic considerations affecting the calculated HOMO–LUMO gap in protein molecules. *J. Phys.: Condens. Matter* **2013**, *25*, 152101.

(9) Dolfen, A. Massively Parallel Exact Diagonalization of Strongly Correlated Systems. 2011.

(10) Plekhanov, E.; Hasnip, P.; Sacksteder, V.; Probert, M.; Clark, S. J.; Refson, K.; Weber, C. Many-Body Renormalization of Forces in F-Electron Materials. *Phys. Rev. B* **2018**, *98*, 075129.

(11) Weber, C.; Amaricci, A.; Capone, M.; Littlewood, P. B. Augmented Hybrid Exact-Diagonalization Solver for Dynamical Mean Field Theory. *Phys. Rev. B* **2012**, *86*, 115136.

(12) O'Regan, D. D.; Hine, N. D. M.; Payne, M. C.; Mostofi, A. A. Linear-Scaling DFT + U With Full Local Orbital Optimization. *Phys. Rev. B* **2012**, *85*, 085107.

(13) O'Regan, D. D. *Optimised Projections for the Ab Initio Simulation of Large and Strongly Correlated Systems*; Springer Berlin Heidelberg: Berlin, Heidelberg, 2012; pp 65–88.

(14) O'Regan, D. D. *Optimised Projections for the Ab Initio Simulation of Large and*

Strongly Correlated Systems; Springer Berlin Heidelberg: Berlin, Heidelberg, 2012; pp 89–123.

(15) Opium - Pseudopotential Generation Project. 2017; `http://opium.sourceforge.net`.

(16) Kerker, G. P. Non-singular atomic pseudopotentials for solid state applications. *J. Phys. C Solid State Phys.* **1980**, *13*, L189–L194.

(17) Kleinman, L.; Bylander, D. M. Efficacious Form for Model Pseudopotentials. *Phys. Rev. Lett.* **1982**, *48*, 1425–1428.

(18) Hamann, D. R. Generalized Norm-Conserving Pseudopotentials. *Phys. Rev. B* **1989**, *40*, 2980–2987.

(19) Rappe, A. M.; Rabe, K. M.; Kaxiras, E.; Joannopoulos, J. D. Optimized Pseudopotentials. *Phys. Rev. B* **1990**, *41*, 1227–1230.

(20) Gonze, X.; Stumpf, R.; Scheffler, M. Analysis of Separable Potentials. *Phys. Rev. B* **1991**, *44*, 8503–8513.

(21) Ramer, N. J.; Rappe, A. M. Designed nonlocal pseudopotentials for enhanced transferability. *Phys. Rev. B* **1999**, *59*, 12471–12478.

(22) Grinberg, I.; Ramer, N. J.; Rappe, A. M. Transferable relativistic Dirac-Slater pseudopotentials. *Phys. Rev. B* **2000**, *62*, 2311–2314.

(23) Weber, C.; O'Regan, D. D.; Hine, N. D. M.; Littlewood, P. B.; Kotliar, G.; Payne, M. C. Importance of Many-Body Effects in the Kernel of Hemoglobin for Ligand Binding. *Phys. Rev. Lett.* **2013**, *110*, 106402.
